# Neutrophil-to-lymphocyte ratios as easy-to-use biomarkers for the diagnosis of active tuberculosis in children and adolescents

**DOI:** 10.3389/fcimb.2026.1743922

**Published:** 2026-02-05

**Authors:** Jinyu Chen, Dongmei Wang, Bin Deng, Chuan Wang, Shenjie Tang, Lei Chen, Qi An

**Affiliations:** 1 Department of Tuberculosis, Public Health Clinical Center of Chengdu, Chengdu, Sichuan, China; 2 Scientific Research and Teaching Department, Public Health Clinical Center of Chengdu, Chengdu, Sichuan, China; 3 Beijing Chest Hospital, Capital Medical University, Beijing, China

**Keywords:** active tuberculosis (ATB), children and adolescents, diagnostic biomarker, logarithmic neutrophil-to-lymphocyte ratio (logNLR), risk stratification

## Abstract

**Background:**

The diagnosis of active tuberculosis (ATB) in children and adolescents is limited by non-specific symptoms, paucibacillary infection, and the low sensitivity of traditional tools. These limitations can lead to delayed treatment and increased complications.

**Methods:**

This retrospective study recruited 1,080 participants. We performed receiver operating characteristic (ROC) curves to evaluate the diagnostic performance of logarithmic neutrophil-to-lymphocyte ratio (logNLR) for ATB infection. We employed logistic regression, restricted cubic spline (RCS), stratified, and interaction analyses to evaluate the association between logNLR and ATB infection.

**Results:**

The logNLR was significantly associated with ATB infection in the adjusted model (OR = 1.38, 95% CI: 1.01-1.88, P = 0.044). The RCS curve indicated a non-linear relationship between logNLR and ATB infection, with the critical threshold of 0.9232. The breakpoint analyses further confirmed that when logNLR<1.379, the indicator logNLR was positively correlated with ATB infection. Stratified analyses showed logNLR was a reliable predictor in males, 0–7 and 15–17 years old, those with *Mycobacterium tuberculosis* (MTB) exposure, and participants with CD4+ T cell counts>414 cells/μL or CD8+ T cell counts>238 cells/μL (all P<0.05). Interaction analyses revealed that children with both CD4+ T cell counts ≤414 cells/μL and MTB exposure had a substantially higher ATB risk (OR = 19.31). Similarly, synergies were observed in combinations of CD4+ T cell counts ≤414 cells/μL with 0–14 years old, and MTB exposure with 0–14 years old.

**Conclusions:**

The logNLR is a simple, low-cost, and effective biomarker for diagnosis of ATB in children and adolescents. The critical threshold and breakpoint of logNLR enable precise risk stratification, providing valuable support for early ATB identification in this population.

## Graphical Abstract

**Figure:**
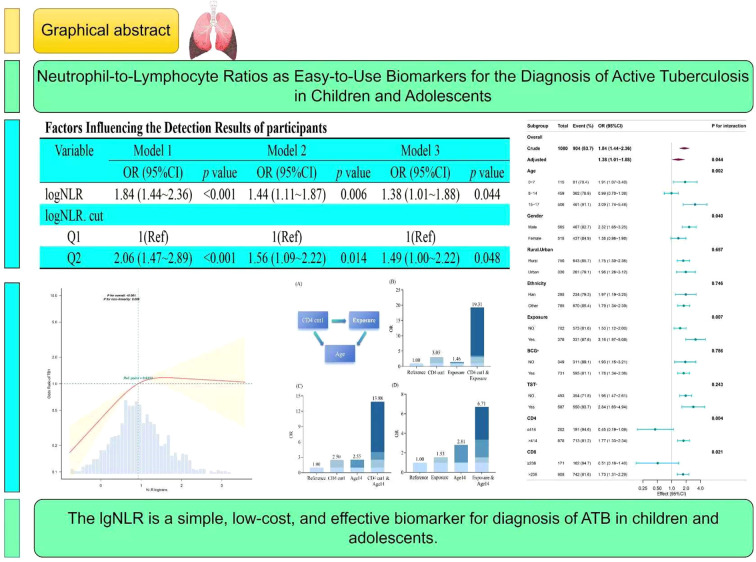

## 1 Introduction

Tuberculosis (TB) is one of the leading causes of infectious disease related mortality worldwide. According to the “Global TB Report 2024”, the World Health Organization (WHO) estimated 10.8 million new TB cases and 1.25 million TB-related deaths in 2023 (World Health Organization, a). Notably, children and adolescents account for 12% of these new cases (World Health Organization, a). However, only 55% of these cases were reported to public health program (World Health Organization, a). The gap in case detection mainly stems from challenges in TB diagnosis. TB in children and adolescents often shows non-specific symptoms, such as fever, cough and weight loss (Inbaraj et al., 2025). Furthermore, the disease in this population is typically paucibacillary, and obtaining adequate sputum samples is difficult (Smith et al., 2023). Traditional diagnostic tools have poor performance in children, as less than 15% of children are sputum acid-fast bacilli smear positive. *Mycobacterium tuberculosis* (MTB) culture which is the “gold standard”, only yields 30%-40% positive results (Smith et al., 2023). In the new version of pediatric TB guidelines issued by WHO in 2022, it is recommended to use the Xpert MTB/RIF Ultra rapid molecular detection as the initial detection of pediatric TB (World Health Organization, b). However, the sensitivity of urine, feces and other non-sputum samples for the Xpert Ultra diagnosis of TB is still not high (Zar et al., 2019; Cox et al., 2022). Both tuberculin skin test (TST) and interferon gamma release assays (IGRAs) show reduced sensitivity in children with immature or compromised immune systems (Nolt and Starke, 2021). The lack of effective diagnostic tools leads to delayed treatment of TB. This delay of ATB diagnosis increases the risk of severe complications, such as miliary TB and tuberculous meningitis (Tedla et al., 2020). It also raises the chance of TB transmission among pediatric populations (Zhang et al., 2023). Globally, 96% of TB-related deaths in individuals younger than 15 years old occur in those who have not initiated treatment (World Health Organization, c; Bijker et al., 2024). Therefore, there is an urgent need for novel diagnostic tools.

In recent years, the neutrophil-to-lymphocyte ratio (NLR), a low-cost, easily calculable marker derived from routine complete blood counts (CBC), has emerged as a promising inflammatory biomarker for TB diagnosis. Multiple studies have validated its utility in distinguishing active TB (ATB) from latent TB infection (LTBI) or other infectious diseases (Abakay et al., 2014; Cursi et al., 2023). These findings based on adults have provided a foundation for applying of the NLR in TB infection. However, the application of NLR has several limitations. First, NLR lacks specificity, as it can be elevated in bacterial infections, malignancies and other conditions (Janka et al., 2023; Ueda et al., 2023). Second, NLR is susceptible to confounding factors like age, BMI and comorbidities (Tang et al., 2022). Third, the diagnostic cut-offs for NLR vary across different studies (Liu et al., 2022; Li et al., 2023). In addition, children and adolescents have immature immune systems and physiologically fluctuating NLR, so adult conclusions cannot be directly extrapolated. However, current pediatric studies on NLR and TB have present notable limitations. For instance, the study of Ma et al. included only 176 TB cases (Ma et al., 2024), and the study of Kissling et al. included just 12 TB cases (Sheng et al., 2025). Moreover, these studies did not adjust the confounding factors, such as age and immune status. The limitations of these results mean they are insufficient to meet clinical needs. Additionally, in clinical datasets, the raw NLR values often exhibit skewed distributions. This skewed distribution may reduce the stability of diagnostic cut-offs across different populations (Zahorec, 2021). Logarithmic transformation of NLR (logNLR) can enhance the consistency and reproducibility of biomarkers in inflammatory diseases (Tu et al., 2023; Man et al., 2025). However, the application of logNLR in the diagnosis of children and adolescents with ATB remains unexplored.

To address the unmet need for effective ATB diagnosis in children and adolescents, this study explored the diagnostic value of logNLR. The study enrolled 904 children and adolescents with ATB and 176 controls with non-ATB group infections (including bacterial pneumonia, non-tuberculous mycobacterial pulmonary disease, and parasitic infection). We collected demographic, clinical, and laboratory data, which included neutrophil and lymphocyte counts, and then applied receiver operating characteristic (ROC) curves, logistic regression analyses, restricted cubic spline (RCS) analyses, stratified analyses, and interaction analyses to assess the association between logNLR and ATB infection in this population. This study first sought to clarify the diagnostic performance of logNLR for children and adolescents with ATB. Moreover, we identified the potential non-linear relationship and critical thresholds between logNLR and ATB infection. Meanwhile, we further explored the stability of logNLR in different subgroups. This study intended to demonstrate logNLR as a simple, low-cost diagnostic biomarker for ATB in children and adolescents.

## 2 Methods

### 2.1 Participants

We conducted a retrospective study on children and adolescents diagnosed with ATB who were hospitalized at the Public Health Clinical Medical Center of Chengdu from April 2022 to December 2024. Ethical approvals for this study were obtained from the Ethics committee of the Public Health Clinical Medical Center of Chengdu (Ethics Approval Number: YJ-K2024-75-01).

The inclusion criteria for the study were children and adolescents aged under 18 years old, covering both those diagnosed with ATB and non-ATB patients. The exclusion criteria were patients with incomplete clinical or laboratory data (such as missing results of blood routine, liver function, or kidney function tests), confirmed infection with the Human Immunodeficiency Virus (HIV) or diagnosis of Acquired Immune Deficiency Syndrome (AIDS), comorbidity with other diseases that may affect study results (including but not limited to cancer and autoimmune diseases), non-active TB, or a history of receiving anti-cytokine therapy and/or other immunosuppressive therapies before hospitalization.

Patients were divided into the ATB group and the non-ATB group based on diagnosis. The ATB group further comprised the Etiologically confirmed TB group (E-TB) and the clinically diagnosed TB group (C-TB). Specifically, the E-TB group included patients who met both positive results in molecular biology tests for MTB or positive MTB culture results, and the presence of radiological manifestations consistent with tuberculosis; the C-TB group consisted of patients who presented with radiological manifestations consistent with tuberculosis (with or without clinical symptoms of tuberculosis) and showed a positive response to anti-tuberculosis treatment. Meanwhile, the non-ATB group included patients who were hospitalized during the same study period and diagnosed with diseases other than tuberculosis, including bacterial pneumonia, non-tuberculous mycobacterial (NTM) lung disease, and parasitic infections.

We obtained the demographic and clinical data from electronic patient records to conduct comparisons between the cases of ATB group and non-ATB group.

### 2.2 Laboratory analysis

Only laboratory data of patients diagnosed during their first hospitalization were collected. For blood routine data, the first test results obtained within 24 hours after admission were recorded. For other laboratory data, the initial test results after admission were collected. The absolute counts of neutrophils, lymphocytes, CD4^+^ T cells and CD8^+^ T cells were collected. The logarithmic neutrophil-to-lymphocyte ratio (logNLR) was defined as the logarithm of the ratio of absolute neutrophil count to absolute lymphocyte count. The method of calculating the NLR, logNLR, MLR, NMLR and NAR are as follows:


$$\text{NLR }=\text{ Neutrocyte }\left(\times 10^{9}/\text{L}\right)\;÷\text{ Lymphocyte }\left(\times 10^{9}/\text{L}\right)$$

$$\begin{array}{c} \text{logNLR }=\text{ lo}{\text{g}}_{e}\left[\text{Neutrocyte }\left(\times 10^{9}/\text{L}\right)\;÷\text{ Lymphocyte }\left(\times 10^{9}/\text{L}\right)\right]\;\\ =\text{ ln }\left[\text{Neutrocyte }\left(\times 10^{9}/\text{L}\right)\;÷\text{ Lymphocyte }\left(\times 10^{9}/\text{L}\right)\right] \end{array}$$

$$\text{MLR }=\text{ Monocyte }\left(\times 10^{9}/\text{L}\right)\;÷\text{ Lymphocyte}\left(\times 10^{9}/\text{L}\right)$$

$$\text{NMLR }=\;\left[\text{Neutrophil }\left(\times 10^{9}/\text{L}\right)\;+\text{ Monocyte }\left(\times 10^{9}/\text{L}\right)\right]\;÷\text{ Lymphocyte }\left(\times 10^{9}/\text{L}\right)$$

$$\text{NAR}=\text{ Neutrocyte }\left(\times 10^{9}/\text{L}\right)\;÷\text{ Albumin }\left(\text{g}/\text{L}\right)$$

### 2.3 Statistical analyses

We used an electronic medical record system for data management (http://clinicaledc.com). This study adopted a total sampling method, and the sample comprised inpatients who met the inclusion and exclusion criteria at the Public Health Clinical Medical Center of Chengdu from April 2022 to December 2024. For the data in this study, the continuous data that do not conform to the normal distribution were expressed as M (Q1, Q3) and analyzed using the rank sum test. The categorical data were described by frequency and percentage (%) and the Chi-square test was used to compare differences between groups. When testing differences between groups, we selected the χ² test or Fisher exact test for categorical variables, the one-way ANOVA test for data with normal distribution, and the Kruskal-Wallis H test for data with skewed distribution.

We observed that the NLR data exhibited skewness, and thus natural logarithmic transformation was performed on the NLR values prior to statistical analysis, with log-transformed NLR (logNLR) used for subsequent statistical testing (**Supplementary Figures S1**). To elucidate the relationship between logNLR and ATB infection, we established logistic regression analyses with the lowest quartile (Q1) as the reference group. Moreover, after adjusting for covariates, we performed the restricted cubic spline (RCS) analyses between logNLR and ATB infection to investigate potential non-linear relationships. We conducted stratified analyses to assess the potential impact of age, gender, ethnicity, rural/urban residence, BCG vaccination, TST result, MTB exposure history, CD4+ T cells and CD8+ T cells on the association between logNLR and ATB infection. We used the relative excess risk due to interaction (RERI), attributable proportion due to interaction (AP) and synergy index (SI) to assess additive interactions. We carried out statistical analyses using R software (http://www.R-project.org, The R Foundation) and Free Statistics software version 2.3. We drew the bar chart using GraphPad Prism version 9.0. For the study subjects, we conducted descriptive statistics, with a significance level set at P<0.05.

## 3 Results

### 3.1 Baseline characteristics of study participants

**Figure 1:**
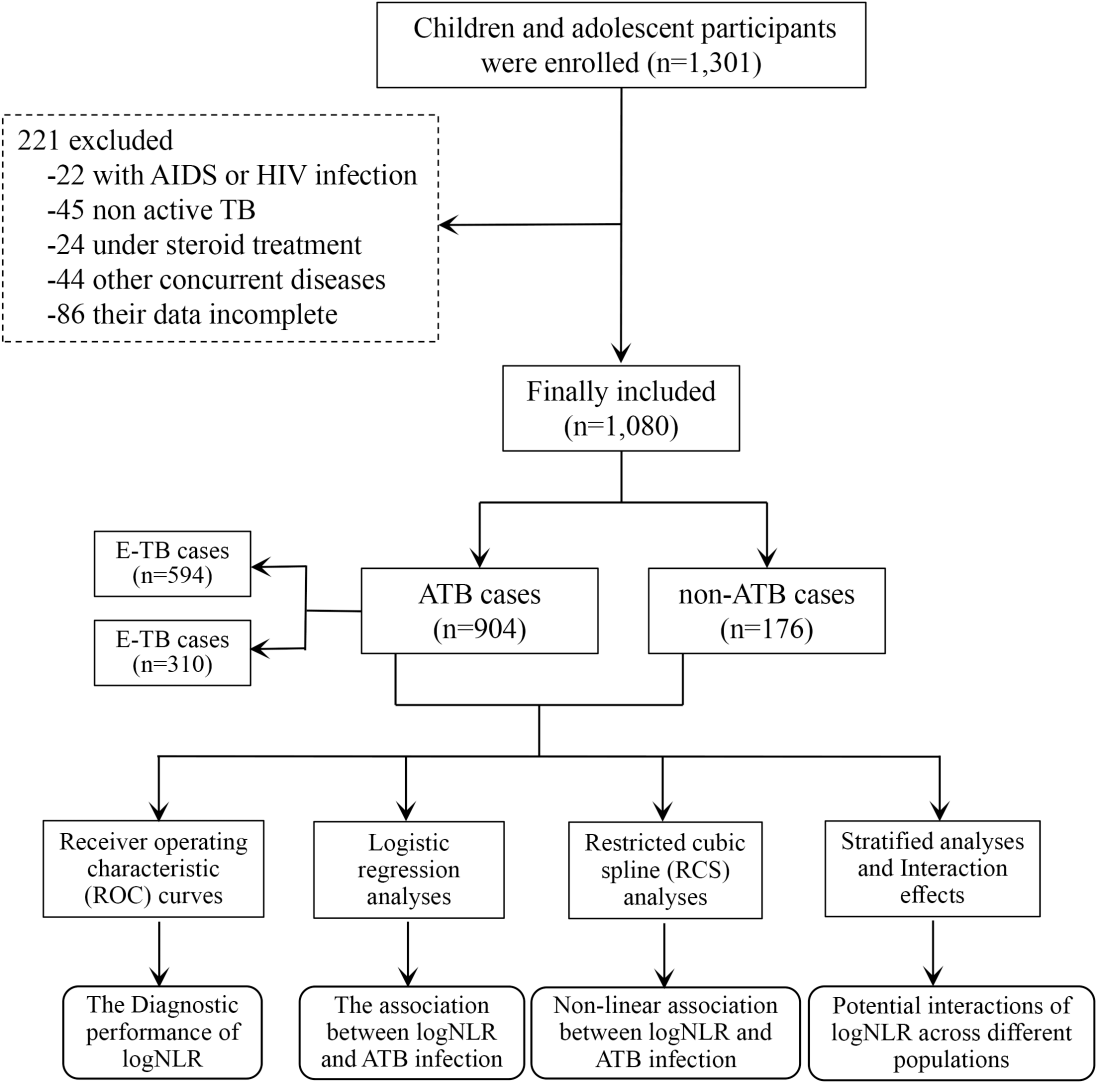
Flowchart of participants in this study. TB, tuberculosis; AIDS, Acquired Immune Deficiency Syndrome; HIV, Human Immunodeficiency Virus; ATB, active tuberculosis.

Between April 2022 and December 2024, a total of 1,301 subjects were collected in the study. Among them, 221 cases (17.0%) were excluded from the analysis according to the exclusion criteria, including 22 cases of AIDS or HIV infection, 45 cases of non-active TB (patients with previous anti-tuberculosis therapy, no symptoms and signs related to tuberculosis, negative tuberculosis etiology, and stable imaging lesions), 24 cases of under steroid treatment, 44 cases with other diseases such as tumors and autoimmune diseases, and 86 cases with incomplete data. Finally, clinical/laboratory data from 1080 patients were included in the final analysis (**Figure 1**). Patients were grouped non-ATB group (n=176 [16.30%]) and ATB group (n=904 [83.70%]). The ATB group further was stratified into E-TB group (n=594) and C-TB group (n=310).

The average age was 13.0 ± 3.7 years old, with 115 (10.6%) participants aged 0–7 years old, 459 (42.5%) participants aged 8–14 years old, and 506 (46.9%) participants aged 15–17 years old. The age distribution differed significantly between ATB group and non-ATB group (P<0.001). Gender showed no significant difference (P = 0.328), with 565 (52.3%) participants were male. Moreover, significant disparities were observed in rural-urban residence (P = 0.006), ethnicity (P = 0.017), MTB exposure status (P = 0.012), BCG vaccination (P<0.001), TST results (P<0.001), CD4+ T cell counts (P<0.001), CD8+ T cell counts (P<0.001), NLR (P<0.001), logNLR (P<0.001), MLR (P<0.001) and NMLR (P<0.001) between ATB group and non-ATB group. In contrast, NAR (P = 0.333) showed no statistically significant differences between ATB group and non-ATB group. In the comparison among the non-ATB, E-TB, and C-TB groups, similar disparities were observed, with significant overall differences (P<0.05) across 13 variables, including age, urban-rural residence, and ethnicity, while gender showed no significant difference (P = 0.572). Detailed information is shown in **Table 1**.

**Table 1:** Baseline demographics of participants.

Variables	Total (n=1080)	non-ATB (n=176)	ATB (n = 904)	P^c^ value	E-TB (n= 594)	C-TB (n=310)	P^d^ value
Age, [years], n (%)				< 0.001			<0.001
0-7	115 (10.6)	34 (19.3)	81 (9.0)		41 (6.9)	40 (12.9)	
8-14	459 (42.5)	97 (55.1)	362 (40.0)		231 (38.9)	131 (42.3)	
15-17	506 (46.9)	45 (25.6)	461 (51.0)		322 (54.2)	139 (44.8)	
Gender, n (%)				0.328			0.572
Male	565 (52.3)	98 (55.7)	467 (51.7)		304 (51.2)	163 (52.6)	
Female	515 (47.7)	78 (44.3)	437 (48.3)		290 (48.8)	147 (47.4)	
Rural/Urban, n (%)				0.006			0.013
Rural	750 (69.4)	107 (60.8)	643 (71.1)		430 (72.4)	214 (69)	
Urban	330 (30.6)	69 (39.2)	261 (28.9)		164 (27.6)	96 (31)	
Ethnicity, n (%)				0.017			<0.001
Han	295 (27.3)	61 (34.7)	234 (25.9)		132 (22.2)	102 (32.9)	
Other	785 (72.7)	115 (65.3)	670 (74.1)		462 (77.8)	208 (67.1)	
Exposure status, n (%)				0.012			0.001
No	702 (65.0)	129 (73.3)	573 (63.4)		358 (60.3)	215 (69.4)	
Yes	378 (35.0)	47 (26.7)	331 (36.6)		236 (39.7)	95 (30.6)	
BCG^a^, n (%)				<0.001			<0.001
–	349 (32.3)	38 (21.6)	311 (34.4)		224 (37.7)	87 (28.1)	
+	731 (67.7)	138 (78.4)	593 (65.6)		370 (62.3)	223 (71.9)	
TST^b^, n (%)				<0.001			<0.001
–	493 (45.6)	139 (79.0)	354 (39.2)		267 (44.9)	87 (28.1)	
+	587 (54.4)	37 (21.0)	550 (60.8)		327 (55.1)	223 (71.9)	
CD4, cells/l, n (%)				<0.001			<0.001
≤414	202 (18.7)	11 (6.2)	191 (21.1)		146 (24.6)	46 (14.8)	
>414	878 (81.3)	165 (93.8)	713 (78.9)		448 (75.4)	264 (85.2)	
CD8, cells/l, n (%)				<0.001			<0.001
≤238	171 (15.8)	9 (5.1)	162 (17.9)		130 (21.9)	32 (10.3)	
>238	909 (84.2)	167 (94.9)	742 (82.1)		464 (78.1)	278 (89.7)	
NLR, Median (IQR)	2.5 (1.7, 3.9)	2.0 (1.2, 2.9)	2.6 (1.8, 4.0)	<0.001	3.0 (1.9, 4.8)	2.2 (1.4, 3.0)	<0.001
logNLR, Median (IQR)	0.9 (0.5, 1.4)	0.7 (0.2, 1.1)	1.0 (0.6, 1.4)	<0.001	1.1 (0.7, 1.6)	0.8 (0.4, 1.1)	<0.001
MLR, Median (IQR)	0.2 (0.2, 0.4)	0.2 (0.1, 0.3)	0.2 (0.2, 0.4)	<0.001	0.3 (0.2, 0.5)	0.2 (0.2, 0.3)	<0.001
NMLR, Median (IQR)	2.7 (1.9, 4.3)	2.2 (1.4, 3.1)	2.9 (2.0, 4.4)	<0.001	3.3 (2.1, 5.2)	2.4 (1.6, 3.3)	<0.001
NAR, Median (IQR)	0.1 (0.1, 0.1)	0.1 (0.1, 0.1)	0.1 (0.1, 0.1)	0.333	0.1 (0.1, 0.1)	0.1 (0.1, 0.1)	<0.001

TB, tuberculosis; ATB, active tuberculosis; E-TB, Etiologically confirmed TB; C-TB, clinically diagnosed TB; BCG, Bacillus Calmette-Guérin; TST, tuberculin skin test; NLR, Neutrophil-Lymphocyte ratio; logNLR, logarithmic Neutrophil-Lymphocyte ratio; MLR, Monocyte-to-Lymphocyte Ratio; NMLR, Neutrophil-and-Monocyte-to-Lymphocyte Ratio; NAR, Neutrophil-Albumin ratio.

BCG^a^: The BCG vaccination status was determined by collecting vaccination history from the guardians of the children and adolescents. Since newborns in China are required to receive BCG vaccination, patients with unknown vaccination status were included in the group with vaccination history. TST^b^: Missing results are defaulted to negative. P^c^: The P value was when the two groups (non-ATB and ATB) were compared. P^d^:The P value was when the three groups (non-ATB, E-TB and C-TB) were compared.

### 3.2 Diagnostic performance of biomarkers

**Figure 2:**
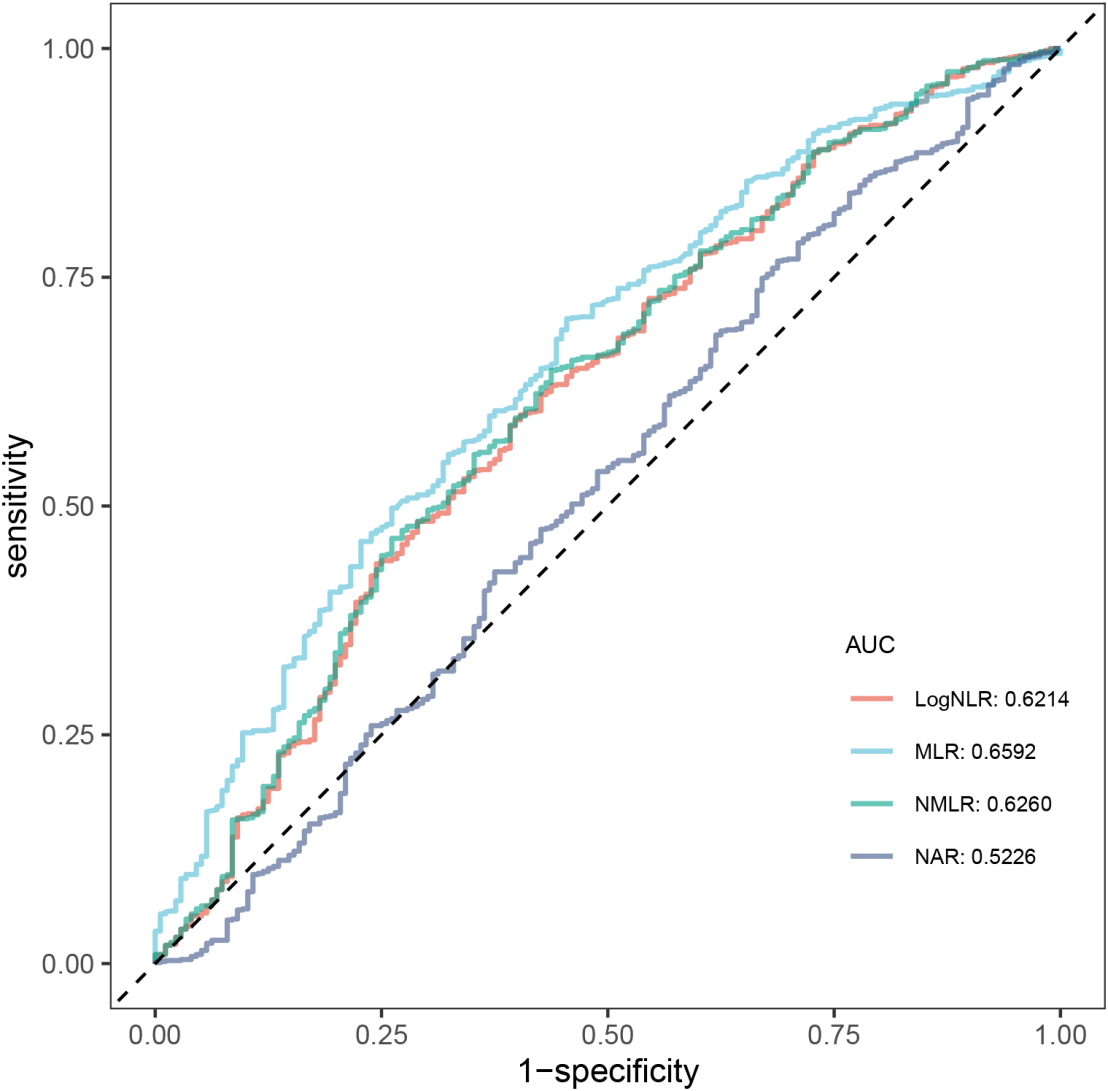
ROC curve analysis of biomarkers between ATB and non-TB. ROC, receiver operating characteristic; ATB, active tuberculosis; TB, tuberculosis; AUC, area under the curve; NLR, Neutrophil-Lymphocyte ratio; logNLR, logarithmic Neutrophil-Lymphocyte ratio; MLR, Monocyte-to-Lymphocyte Ratio; NMLR, Neutrophil-and-Monocyte-to-Lymphocyte Ratio; NAR, Neutrophil-Albumin ratio.

The area under the curve (AUC) was calculated to compare the accuracy of logNLR with that of other biomarkers (MLR, NMLR and NAR) in predicting the risk of ATB infection. **Figure 2** presented ROC curves assessing the predictive performance of logNLR and other biomarkers for ATB infection. The AUCs of logNLR, MLR, NMLR and NAR were 0.6214, 0.6592, 0.6260 and 0.5226. **Table 2** further provided the sensitivity, specificity, PPV, NPV, threshold, and accuracy of each biomarkers.

**Table 2:** Comparison of AUC values between logNLR and other biomarkers.

Variable	AUC	Threshold	Specificity	Sensitivity	Accuracy	PPV	NPV
logNLR	0.6214	0.83	0.60	0.59	0.60	0.88	0.22
MLR	0.6592	0.80	0.55	0.70	0.68	0.89	0.26
NMLR	0.6260	0.82	0.56	0.65	0.63	0.88	0.24
NAR	0.5226	0.84	0.31	0.77	0.69	0.85	0.21

AUC, area under the curve; NLR, Neutrophil-Lymphocyte ratio; logNLR, logarithmic Neutrophil-Lymphocyte ratio; MLR, Monocyte-to-Lymphocyte Ratio; NMLR, Neutrophil-and-Monocyte-to-Lymphocyte Ratio; NAR, Neutrophil-Albumin ratio; PPV, Positive Predictive Value; NPV, Negative Predictive Value.

### 3.3 The association between logNLR and ATB infection

**Table 3** presented the association between logNLR and ATB infection explored using logistic regression analyses. Model 1 (crude model) revealed a positive correlation between logNLR and ATB infection (OR = 1.84, 95% CI: 1.44-2.36, P<0.001). In Model 2 (adjusted for Age, Gender, Ethnicity, Rural/Urban), logNLR still exhibited a significant positive association (OR = 1.44, 95% CI: 1.11-1.87, P = 0.006). In Model 3 (further adjusted for BCG, TST, Exposure, CD4, and CD8 on the basis of Model 2), logNLR remained significantly positively associated (OR = 1.38, 95% CI: 1.01-1.88, P = 0.044). After multivariate adjustment, each one-unit increase in IogNLR was associated with a 44% (Model 2) and 38% (Model 3) increased odds of the outcome, respectively.

**Table 3:** Factors influencing the detection results of participants.

Variable	Model 1		Model 2		Model 3	
	OR (95%CI)	P value	OR (95%CI)	P value	OR (95%CI)	*P* value
logNLR	1.84 (1.44-2.36)	<0.001	1.44 (1.11-1.87)	0.006	1.38 (1.01-1.88)	0.044
logNLR. cut						
Q1	1(Ref)		1(Ref)		1(Ref)	
Q2	2.06 (1.47-2.89)	<0.001	1.56 (1.09-2.22)	0.014	1.49 (1.00-2.22)	0.048

Model 1: Crude.

Model 2: Adjust: Age, Gender, Ethnicity, Rural/Urban.

Model 3: Adjust: Age, Gender, Ethnicity, Rural/Urban, BCG^a^, TST^b^, Exposure, CD4, CD8.

When logNLR was dichotomized into two groups, Q2, which had high levels of logNLR, demonstrated a significantly increased risk (OR = 2.06, 95% CI: 1.47-2.89, P<0.001) of ATB infection compared with Q1 (the reference group) in Model 1. Similarly, after adjusting for all covariates (Model 3), Q2 continued to have a significantly increased risk (OR = 1.49, 95% CI: 1.00-2.89, P = 0.048) compared with Q1. Compared to the Q1, participants in the Q2 group of logNLR had 49% (Model 3) higher odds of the outcome after adjustment.

### 3.4 Nonlinear association between logNLR and ATB infection

**Figure 3:**
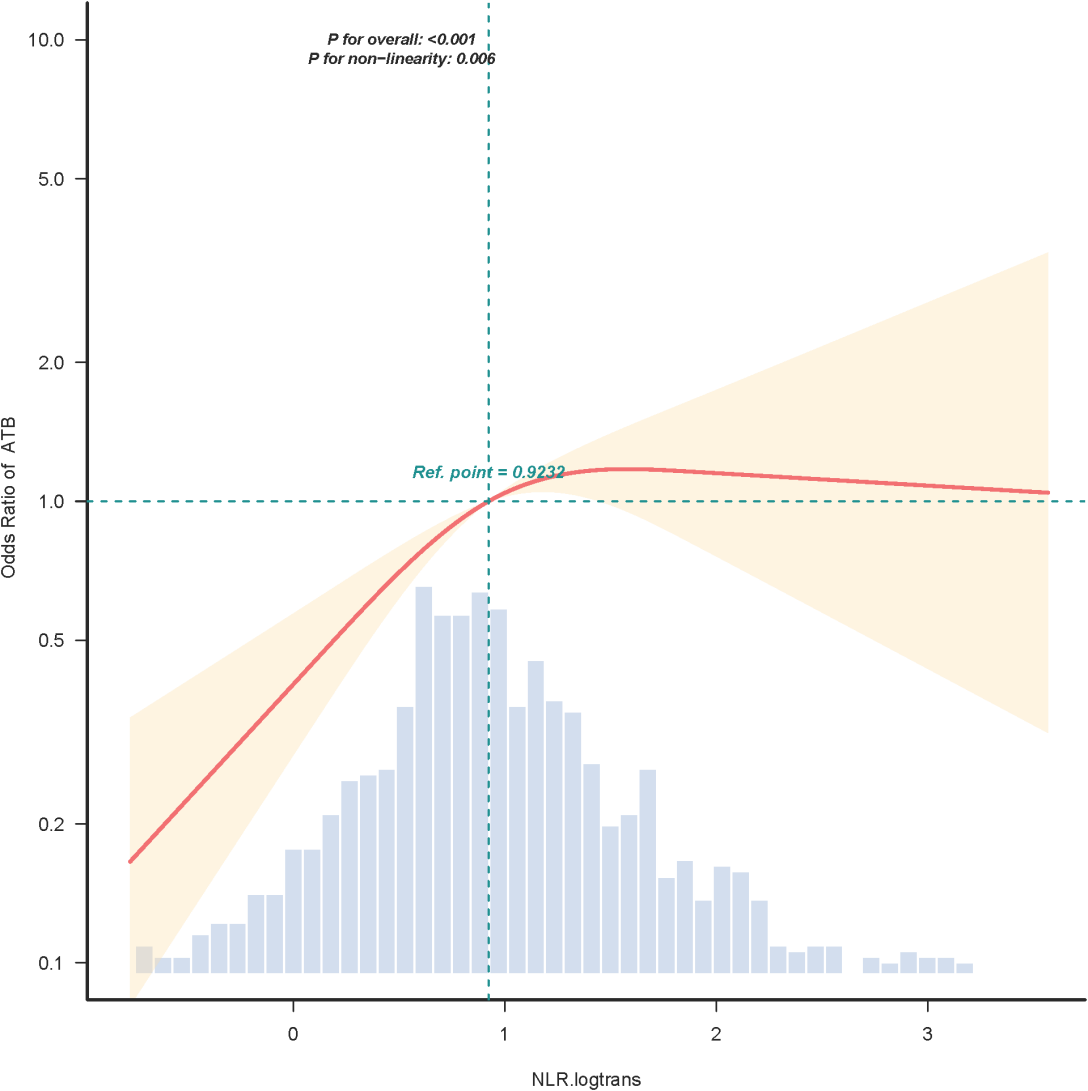
RCS curve of the relationship between logNLR and ATB infection. The OR is represented by the orange line and the shaded part represents the 95% CI. RCS, restricted cubic spline; OR, odds ratio; CI, confidence interval; logNLR, logarithmically transformed neutrophil-to-lymphocyte ratio; ATB, active tuberculosis.

The results of the restricted cubic spline (RCS) curve in **Figure 3** manifested that there was a remarkable overall trend between logNLR and ATB infection risk (P for overall <0.001). In the model adjusting for all covariates according to Model 3 (Age, Gender, Ethnicity, Rural/Urban, BCG, TST, Exposure, CD4, CD8), it was detected that the two may be non-linearly related (P-non-linear=0.006). The critical value of logNLR was approximately 0.9232. When logNLR<0.9232, OR increased with the elevation of logNLR, indicating that logNLR was a risk factor for ATB infection; when logNLR>0.9232, OR tended to stabilize or slightly decrease, suggesting the stabilizing of its risk effect.

**Table 4** presented the breakpoint analyses of the association between logNLR and ATB infection. The estimated breakpoint of this analysis was 1.397. When logNLR<1.397, there was a strong positive association between logNLR and ATB infection (OR = 2.796, 95% CI: 1.96-3.99, P<0.001). This positive association indicated that an increase in logNLR significantly elevated the risk of ATB infection in children and adolescents. Conversely, when logNLR>1.397, the association between logNLR and ATB infection weakened substantially (OR = 0.754, 95% CI: 0.291-1.951, P = 0.5598), and this weakened association showed no statistical significance. Additionally, the likelihood ratio test result (P = 0.016) confirmed the significant non-linear nature of the relationship between logNLR and ATB infection.

**Table 4:** Breakpoint analyses of the logNLR and ATB infection relationship.

Item	Breakpoint/OR (95%CI)	P value
Estimate_Breakpoint	1.397 (1.381-1.412)	NA
logNLR<1.397	2.796 (1.96-3.99)	<0.001
logNLR≥1.397	0.754 (0.291-1.951)	0.5598
Likelihood Ratio test	–	0.016

### 3.5 Stratified analyses between logNLR and ATB infection

**Figure 4:**
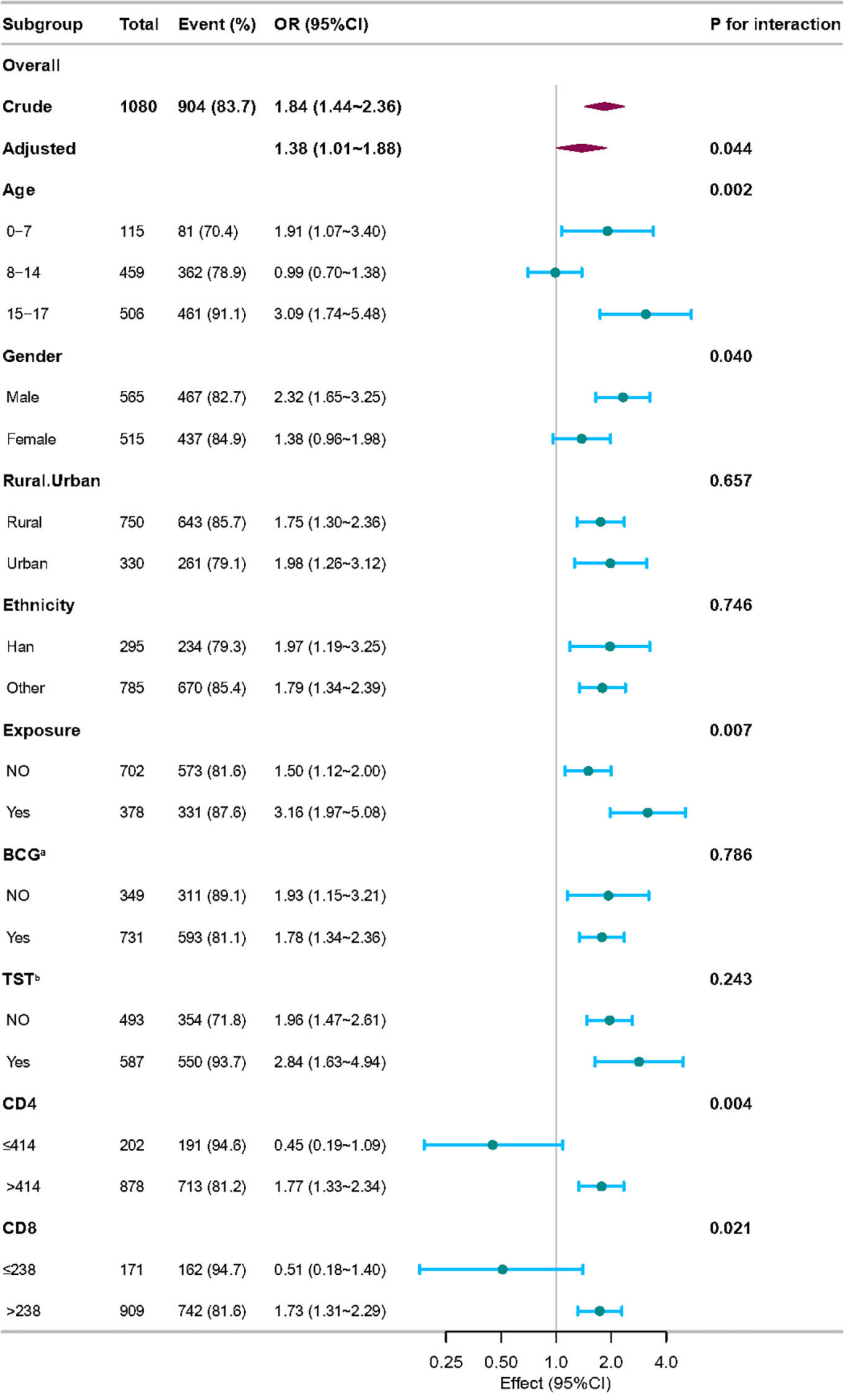
Stratified analyses for the association between logNLR and ATB infection. P for interaction indicated adjusting for Age, Gender, Ethnicity, Rural/Urban, BCG^a^, TST^b^, Exposure, CD4, CD8. OR, odds ratio; CI, confidence interval; logNLR, logarithmically transformed neutrophil-to-lymphocyte ratio; ATB, active tuberculosis.

To assess potential disparities across these populations, we conducted stratified analyses based on Model 3 to investigate the relationship between logNLR and ATB infection in different populations (**Figure 4**). After adjusting for all confounding factors for interaction testing, the results demonstrated that the subgroups of age, gender, exposure, CD4, CD8 remarkably influenced the relationship between logNLR and ATB infection (P for interaction <0.05). The results unveiled that in populations with age of 0–7 and 15–17 years old, male, with MTB exposure history, CD4+ T cell counts>414 cells/μL, CD8+ T cell counts>238 cells/μL, the risk of ATB infection was positively correlated with logNLR (OR>1, P<0.05). For example, participants with age of 15–17 years old had a significantly higher risk of ATB infection (OR: 3.09, 95% CI: 1.74–5.48, P for interaction<0.05).

### 3.6 Interaction effects

**Figure 5:**
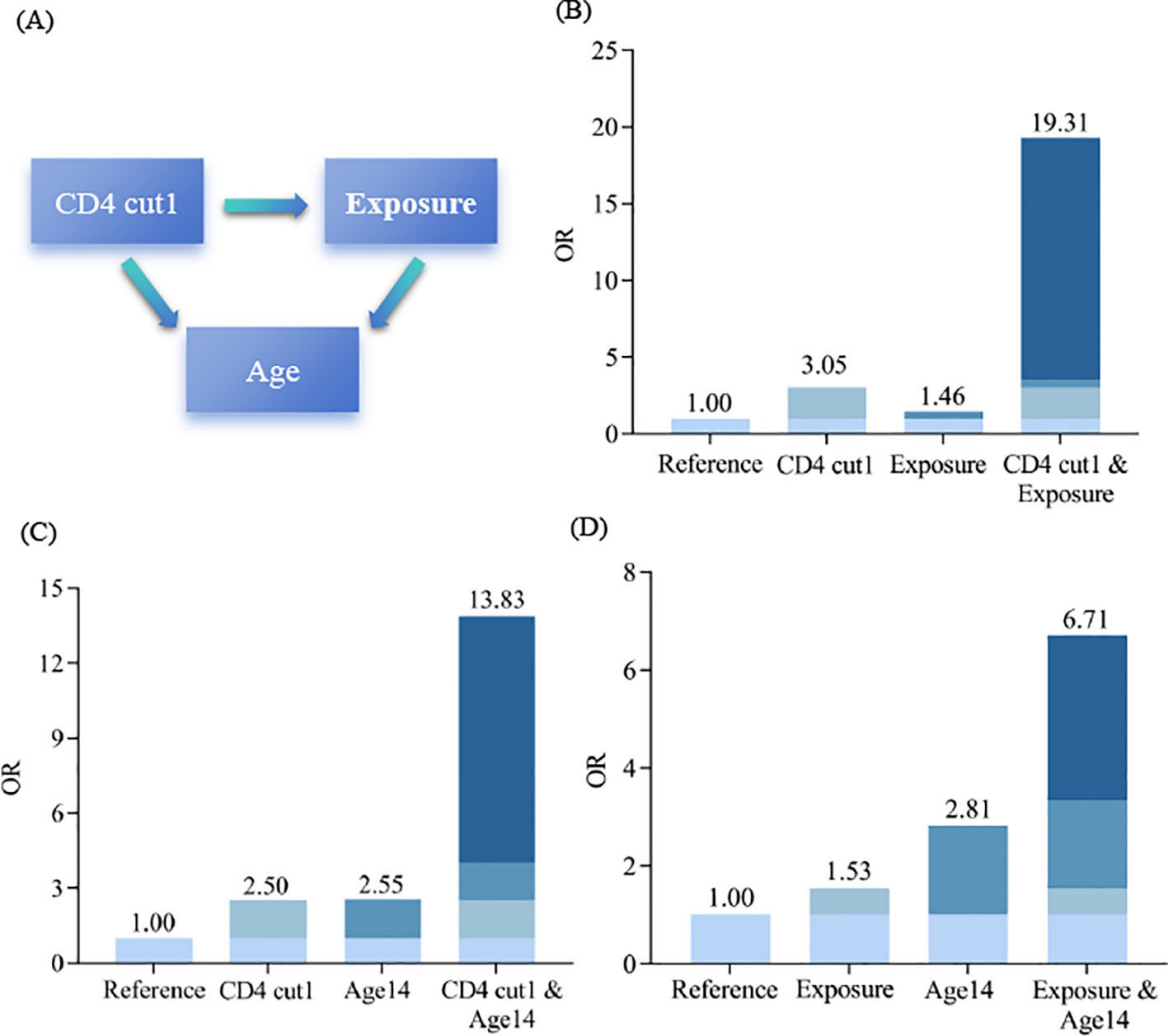
Interaction effects between identified factors on ATB infection risk. **(A)** Schematic diagram of interaction effects among CD4+ T cell count cut-off (414 cells/μL), MTB exposure history, and age. **(B)** Infection effects between CD4+ T cell count ≤414 cells/μL and MTB exposure history. **(C)** Infection effects between CD4+ T cell count ≤414 cells/μL alone and age 0–14 years old. **(D)** Infection effects between MTB exposure history and age 0–14 years.

Our analyses of the interactions between the identified factors regarding the risk of ATB infection were presented in **Figure 5**. For the interaction between CD4+ T cell counts (≤414 cells/μL) and MTB exposure history (YES), CD4+ T cell counts ≤ 414 cells/μL alone was significantly associated with increased ATB infection risk (OR = 3.05, 95% CI: 1.55-6.01, P<0.001), and MTB exposure history alone was marginally significantly associated with elevated ATB risk (OR = 1.46, 95% CI: 1.00-2.12, P = 0.05). Patients with both CD4+ T cell counts ≤ 414 cells/μL and MTB exposure history had a substantially higher ATB risk vs. the reference group (OR = 19.31, 95% CI: 2.66-140.15, P<0.001), and this combined OR (19.31) numerically exceeded the sum of the two individual OR (3.05 + 1.46 = 4.51). Similarly, patients with both CD4+ T cell counts ≤ 414 cells/μL and 0–14 years old had a substantially higher ATB risk vs. the reference group (OR = 13.88, 95% CI: 4.34-44.40, P<0.001). Patients with both 0–14 years old and MTB exposure history also showed a substantially higher ATB risk vs. the reference group (OR = 6.71, 95% CI: 3.17-14.19, P<0.001).

## 4 Discussion

The interaction between innate and adaptive immune responses to MTB invasion is the potential mechanism that connects NLR to ATB infection (Mayer-Barber and Barber, 2015). Neutrophils serve as the first innate immune cells to infiltrate infection sites. When the host is challenged by external pathogens, neutrophils rapidly undergo chemotaxis, accumulate at the infected site, and contribute to the host’s defense against microbial invasion (Phillipson and Kubes, 2019). However, excessive neutrophils activation can induce tissue damage and trigger the release of pro-inflammatory cytokines (e.g., IL-1β, TNF-α), thereby disrupting immune homeostasis (Kolaczkowska and Kubes, 2013). Lymphocytes, particularly CD4+ and CD8+ T cells, are indispensable for adaptive immunity against MTB. During ATB infection, their counts often decline, which may favor MTB proliferation within the host (Wang et al., 2023). The NLR provides a more precise depiction of the dynamic balance between immune cells. The elevation of NLR indicates either increased neutrophil levels or reduced lymphocyte counts. Logarithmic transformation (logNLR) normalizes the skewed distribution of raw NLR values. As demonstrated by Zahorec in study on inflammatory biomarkers (Zahorec, 2021), this transformation enhances the reliability of NLR as a biomarker by minimizing variability associated with factors such as age and comorbidities.

Our study indicated that MLR (AUC = 0.6592) had slightly better diagnostic value than logNLR, while NMLR (AUC = 0.6260) and logNLR (AUC = 0.6214) demonstrated comparable diagnostic performance. In contrast, NAR (AUC = 0.5226) showed no effective discriminatory value. Notably, the ROC curves of MLR and logNLR exhibited highly similar trends, with close sensitivity and specificity performances. Although MLR showed slightly superior diagnostic efficacy, this advantage was minimal. Our study also confirmed that logNLR holds significant diagnostic value for ATB in children and adolescents even after adjusting for multiple confounding factors. The logistic regression results showed that in the fully adjusted model (Model 3), each 1-unit increase in logNLR was associated with a 38% higher risk of ATB. When logNLR was divided into two groups, the high logNLR group (Q2) still had a 49% higher risk of ATB than the low logNLR group (Q1). These findings align with prior pediatric studies. To discriminate between LTBI, ATB disease and other infectious diseases, Cursi et al. conducted a multicenter study involving 649 children, of whom 77 were diagnosed with ATB (Cursi et al., 2023). They study reported that NLR, with the cut-off of 1.5 and AUC of 0.72, had the sensitivity of 61% and specificity of 79%. Kissling et al. analyzed 389 children in Switzerland, 25 of whom had TB disease. They found that NLR was significantly elevated in children with TB disease compared to those with non-TB lower respiratory tract infections (nTB-LRTI). The cut-off value was 0.75, with an AUC of 0.82, sensitivity of 88%, and specificity of 71% (Kissling et al., 2023). Similarly, to compare severe TB (STB) and bacterial community-acquired pneumonia (CAP), Ma et al. conducted a study of 235 children, including 60 with STB, and found that NLR was an independent risk factor for STB. They also developed a predictive nomogram model with an AUC of 0.867, which demonstrated good discriminatory performance (Ma et al., 2024). However, unlike previous pediatric studies with small sample sizes, our study included 1,080 participants, including 904 ATB cases. The large sample size makes statistical results more scientific and reduces the bias caused by random variation in small samples. More importantly, our study accounted for key confounding variables (including age, gender, ethnicity, rural/urban, BCG vaccination status, TST results, exposure status and CD4+/CD8+ T cell counts) that had been overlooked in other pediatric investigations. For example, most newborns in China have been administered BCG vaccination, may influence immune markers including NLR (Guo et al., 2023). CD4+/CD8+ T cell counts reflect immune function, which is particularly important for children with immature immune systems (Sarfas et al., 2021). The inclusion of these covariates makes the association between logNLR and ATB more reliable.

Moreover, our study identified the non-linear relationship between logNLR and ATB infection risk and determined critical thresholds. The RCS analyses showed that the relationship between logNLR and ATB was non-linear (P for non-linearity=0.006), with a critical value of 0.9232. The breakpoint analyses further confirmed a breakpoint of 1.397. To the best of our knowledge, this non-linear pattern has not been reported in previous pediatric ATB studies and may be related to the physiological characteristics of pediatric immune systems. In children and adolescents with mild to moderate inflammation, elevated neutrophil counts or reduced lymphocyte counts drive a substantial rise in logNLR, exhibiting a strong linkage to the risk of ATB infection. The initial steep increase in risk up to logNLR=0.9232 may reflect the early activation of innate immunity and neutrophil recruitment in response to MTB infection (Phillipson and Kubes, 2019). However, when inflammation is severe (logNLR>1.397), the immune system may enter a state of “exhaustion” (Kolaczkowska and Kubes, 2013). The excessive neutrophil activation may cause tissue damage, and lymphocyte counts may be further suppressed, leading to irrelevance between logNLR and ATB infection. These thresholds (0.9232 and 1.397) can help clinicians stratify risk: children with logNLR<0.9232 may have a low ATB infection risk, while those with 0.9232≤logNLR ≤ 1.397 require priority evaluation for ATB.

Furthermore, stratified analyses revealed that logNLR as a biomarker for the diagnosis of ATB infection varies across subgroups. The results showed that logNLR was a significant positive predictor of ATB in children aged 0–7 and 15–17 years old, males, those with MTB exposure history, and those with CD4+ T cell counts>414 cells/μL or CD8+ T cell counts>238 cells/μL (all P<0.05). Among these, children aged 0–7 years old (OR: 1.91, 95% CI: 1.07-3.40) and adolescents aged 15–17 years old (OR: 3.09, 95% CI: 1.74-5.48) showed a stronger association, which may be attributed to immune responses. For young children, the number of CD4+ T cells in the adaptive immune system is low, and their overall immune function is immature. In young school-aged children, the numbers of CD4+ T cells and CD8+ T cells in the adaptive immune system are stable, and their immune function is relatively mature and stable. Adolescents are in the stage of immune system remodeling, where the activation state of CD4+ T cells is abnormal (e.g., increased proportion of HLA-DR+CD4+ T cells), and hormonal changes during puberty disrupt the immune balance (Donald et al., 2010; Basu Roy et al., 2019; Venturini et al., 2019). Notably, logNLR had no significant predictive effect in children with CD4+ T cell counts ≤ 414 cells/μL or CD8+ T cell counts ≤ 238 cells/μL (P>0.05). This suggests that in children and adolescents with impaired cellular immunity (e.g., due to malnutrition or underlying immune diseases), logNLR may not be a reliable biomarker—likely because severe immune suppression disrupts the balance between neutrophils and lymphocytes, weakening the correlation between logNLR and ATB infection. Clinicians should therefore combine other diagnostic tools (e.g., Xpert MTB/RIF) when evaluating ATB in immune compromised children. This study also explored the additive interaction between logNLR and other factors (CD4+ T cell counts, MTB exposure history and age). Children with both CD4+ T cell counts ≤ 414 cells/μL and MTB exposure had a much higher ATB infection risk (OR = 19.31) than those with either factor alone. The impaired cellular immunity (low CD4+ T cells) reduces host defense, and MTB exposure increases pathogen exposure. The combination of these two factors may enhance the susceptibility to ATB infection, thereby affecting the role of logNLR as a diagnostic marker for ATB. For those aged 0–14 years, immature immunity plus MTB exposure or low CD4+ T cells may further weaken anti-MTB capacity, amplifying ATB infection risk.

The data in this study were derived from a hospitalized population, and future research should expand to outpatient and primary-care settings to validate logNLR’s utility in these contexts, where early pediatric ATB screening is crucial. There is an urgent need, particularly in resource-limited areas, for a diagnostic tool that balances speed, accuracy, and operational feasibility to address the pediatric ATB diagnostic bottleneck (Basu and Chakraborty, 2025). Derived from routine complete blood count (CBC), logNLR is a biomarker that requires no specialized equipment or technical expertise, enabling rapid and low-cost preliminary assessment. This stands in contrast to advanced diagnostic tools, such as Xpert MTB/RIF Ultra or AI-based imaging, which remain difficult to implement in low-resource, high-burden settings due to their reliance on specialized infrastructure (Basu and Chakraborty, 2025). Thus, logNLR may serve as a simple, cost-effective triage tool. In primary-care settings, logNLR may serve as a triage tool to prioritize children with specific logNLR value ranges for further confirmatory testing, thereby reducing unnecessary referrals and accelerating treatment initiation. For outpatients with mild or nonspecific symptoms, logNLR may offer a low-cost screening option to rule out high ATB risk. However, this study has several limitations. First, it is a single-center retrospective study, which may introduce selection bias (e.g., all participants were hospitalized patients, excluding outpatients with mild ATB). Multicenter prospective studies are needed to validate the results in more diverse populations. Second, the non-ATB comparator group in this study did not include patients with viral respiratory infections, inflammatory non-infectious conditions, and healthy controls. Viral respiratory infections are highly prevalent among children and adolescents, and share similar clinical manifestations with ATB, rendering them a critical differential diagnosis in clinical practice. Inflammatory non-infectious conditions are characterized by systemic immune activation, which disrupts the balance between neutrophils and lymphocytes, and elevates NLR. This may result in false-positive outcomes when using logNLR for ATB diagnosis. Furthermore, healthy controls are essential for establishing a baseline logNLR range in the pediatric population, which enables the determination of whether the elevated logNLR is ATB-specific. Third, this study did not explore the dynamic changes in logNLR during anti-TB treatment. Therefore, it is unclear whether logNLR can be used to monitor treatment response. To address this limitation, prospective studies should track logNLR changes over treatment.

## 5 Conclusions

In conclusion, logNLR is a simple, low-cost, and effective biomarker for diagnosing ATB in children and adolescents, with its utility being particularly notable in males, 0–7 and 15–17 years old, those with MTB exposure history, and those with normal CD4+/CD8+ T cell counts. The critical thresholds (0.9232 and 1.397) provide a precise reference for clinical risk stratification. Although logNLR has limitations in immune compromised children, it still has important clinical value as an auxiliary diagnostic tool. Future research should focus on validating logNLR in external cohorts and multicenter populations, as well as exploring its combination with other biomarkers (e.g., NMLR, MLR) to further improve diagnostic accuracy. Additionally, it should investigate the utility of logNLR in monitoring treatment response.

## Data Availability

The original contributions presented in the study are included in the article/**Supplementary Material**. Further inquiries can be directed to the corresponding authors.
