# Supplementary figures and images for: Neutrophil-to-lymphocyte ratios as easy-to-use biomarkers for the diagnosis of active tuberculosis in children and adolescents
Source: Front Cell Infect Microbiol. 2026 Feb 5;16:1743922. doi: 10.3389/fcimb.2026.1743922 (PMC12916635; doi:10.3389/fcimb.2026.1743922)

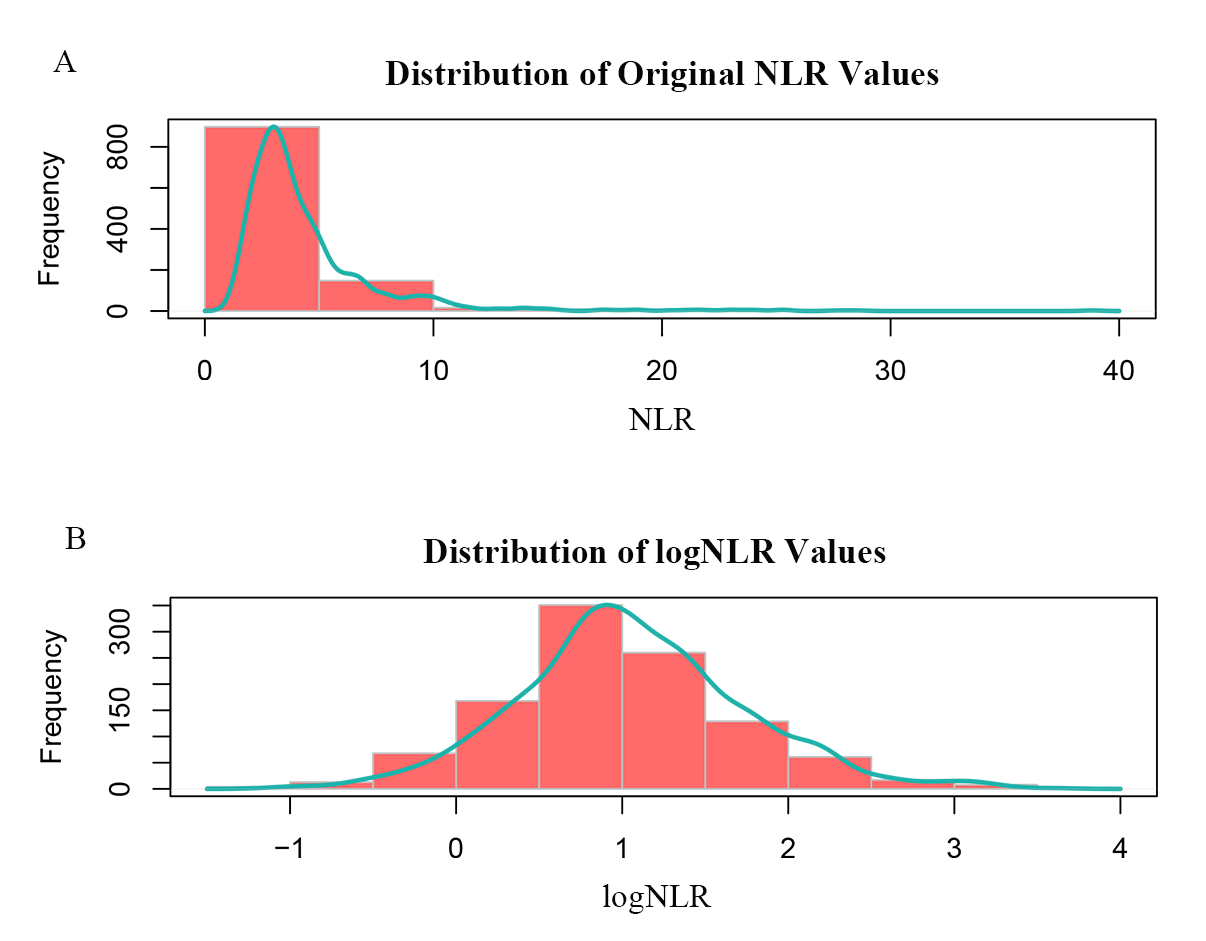

Supplement: Supplementary file 1 [file Image1.tif]
